# Dichlorido{*N*′-[phen­yl(pyridin-2-yl-κ*N*)methyl­idene]isonicotinohydrazide-κ^2^ *N*′,*O*}zinc

**DOI:** 10.1107/S1600536813001281

**Published:** 2013-01-19

**Authors:** Adama Sy, Moussa Dieng, Ibrahima Elhadj Thiam, Mohamed Gaye, Pascal Retailleau

**Affiliations:** a Département de Chimie, Faculté des Sciences et Techniques, Université Cheikh Anta Diop, Dakar, Senegal; b ICSN-CNRS, Laboratoire de Cristallochimie, 1 Avenue la Terasse, 91198 Gift sur Yvette, France

## Abstract

The title compound, [ZnCl_2_(C_18_H_14_N_4_O)], crystallizes with two mol­ecules in the asymmetric unit, which differ in the tautomeric (neutral and zwitterionic) forms of the coordin­ating organic ligand. In both mol­ecules, the Zn^II^ atom adopts a distorted square–pyramidal geometry by two N and one O atoms of the Schiff base ligand and two Cl atoms acting as monodentate chloride anions. The crystal packing is stabilized by N—H⋯N and N—H⋯Cl hydrogen bonds, forming a two-dimensional network parallel to the *ac* plane.

## Related literature

For related structures: see: Addison *et al.* (1984 ▶); Despaigne *et al.* (2009 ▶).
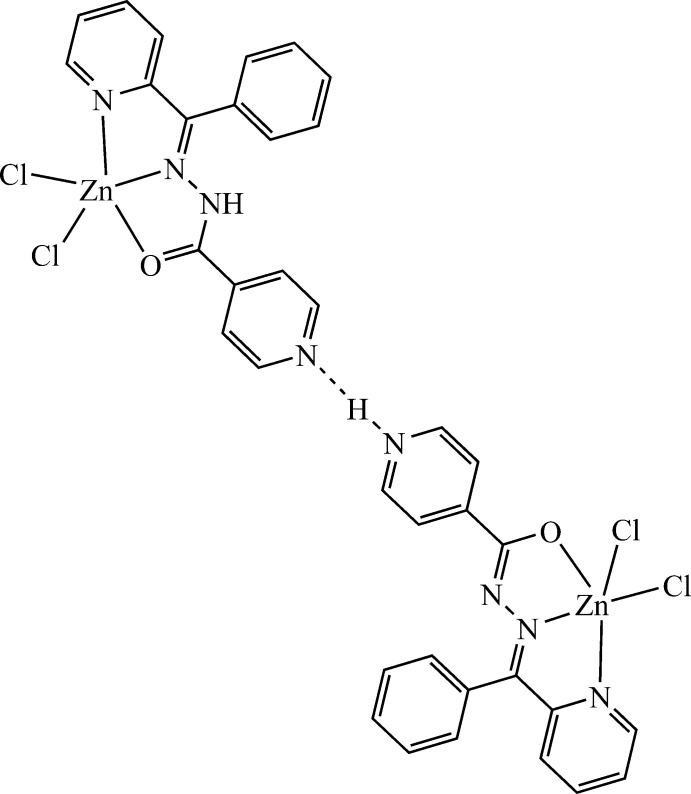


## Experimental

### 

#### Crystal data

[ZnCl_2_(C_18_H_14_N_4_O)]
*M* *_r_* = 438.60
Triclinic, 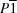

*a* = 11.517 (3) Å
*b* = 13.248 (2) Å
*c* = 13.459 (1) Å
α = 91.130 (5)°
β = 104.708 (4)°
γ = 109.798 (4)°
*V* = 1856.2 (6) Å^3^
*Z* = 4
Mo *K*α radiation
μ = 1.63 mm^−1^
*T* = 293 K
0.45 × 0.22 × 0.18 mm

#### Data collection

Nonius KappaCCD diffractometer
Absorption correction: multi-scan (*ABSCOR*; Rigaku, 2009 ▶) *T*_min_ = 0.379, *T*_max_ = 0.745
15524 measured reflections
6674 independent reflections
4122 reflections with *I* > 2σ(*I*)
*R*_int_ = 0.040

#### Refinement

*R*[*F* ^2^ > 2σ(*F* ^2^)] = 0.042
*wR*(*F* ^2^) = 0.102
*S* = 0.97
6668 reflections
476 parameters
1 restraint
H atoms treated by a mixture of independent and constrained refinement
Δρ_max_ = 0.32 e Å^−3^
Δρ_min_ = −0.42 e Å^−3^

### 

Data collection: *CrystalClear-SM Expert* (Rigaku, 2009 ▶); cell refinement: *CrystalClear-SM Expert*; data reduction: *CrystalClear-SM Expert*; program(s) used to solve structure: *SHELXS97* (Sheldrick, 2008 ▶); program(s) used to refine structure: *SHELXL97* (Sheldrick, 2008 ▶) and *CRYSTALBUILDER* (Welter, 2006 ▶); molecular graphics: *PLATON* (Spek, 2009 ▶); software used to prepare material for publication: *SHELXL97*.

## Supplementary Material

Crystal structure: contains datablock(s) I, global. DOI: 10.1107/S1600536813001281/kq2001sup1.cif

Structure factors: contains datablock(s) I. DOI: 10.1107/S1600536813001281/kq2001Isup2.hkl

Additional supplementary materials:  crystallographic information; 3D view; checkCIF report

## Figures and Tables

**Table 1:** Hydrogen-bond geometry (Å, °)

*D*—H⋯*A*	*D*—H	H⋯*A*	*D*⋯*A*	*D*—H⋯*A*
N5—H5*N*⋯Cl2	0.85 (2)	2.46 (2)	3.250 (3)	156 (4)
N4—H4*N*⋯N8^i^	0.98 (4)	1.78 (4)	2.749 (4)	170 (4)

Symmetry code: (i) 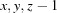.

**Fig. 1.**
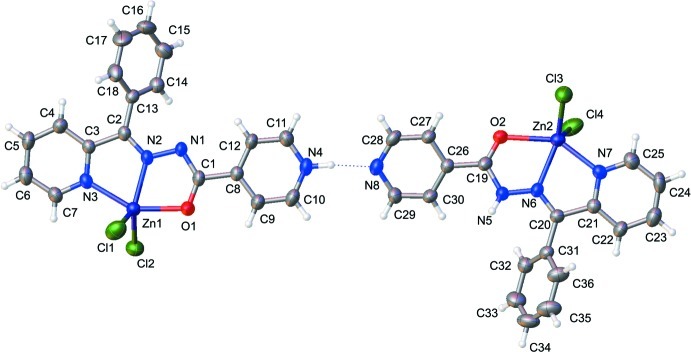
An ORTEP view of the title compound, showing the atom-numbering scheme. Displacement ellipsoids are plotted at the 50% probability level.

## supplementary crystallographic information

### Comment

The title compound crystallizes with two different molecules in the asymmetric unit (Fig. 1), which differ from two tautomeric forms of the Schiff base. The ligand molecules coordinate respectively to one of the zinc(II) atom in a tridentate manner in his hydrazide form and to the other zinc(II) center in the tautomeric form where the nitrogen atom of the uncoordinated pyridine ring is protonated by the proton atom from the nitrogen of the hydrazide moiety. In both cases the coordination manner creates two five-membered chelate metalla rings. The two molecules in the asymmetric unit interact *via* hydrogen bond N—H···N between the two uncoordinated pyridine rings. The bond between Zn center and the pyridine nitrogen are slightly (2.200 (3) and 2.131 (3) Å) different to the two other Zn–N bonds (2.078 (3) and 2.136 (3)). These distances are comparable to those found in the complex [Zn(H_2_BzpClPh)Cl_2_] (H_2_BzpClPh is 2-benzoylpyridine-*para*-chloro-phenylhydrazone) (Despaigne *et al.*, 2009). In the complex, significant variations were observed for the C—O and Zn—O bond distances in the two hydrazone moieties. The C1—O1 bond distance of 1.252 (4) Å is in accordance with a higher single bond character while the C19—O2 bond distance of 1.217 (4) Å is in accordance with a double bond character. The Zn2—O2 bond distance is 2.248 (2) Å while the Zn1—O1 length is 2.127 Å. The decreasing of the length is in accordance with the presence of a negative charge at the oxygen atom O1, which increases the strength of the Zn1—O1 bond. The largest angles around the Zn(II) centers (<β>: N3—Zn1—O1 = 148.50 (11) ° and <β> N7—Zn2—O2 = 114.79 (11) °) are slightly larger than the second-largest ones (<α>: N2—Zn1—Cl2 = 128.30 (10)and <α>: N6—Zn2—Cl3 = 133.10 (10) °). Since the distortion (τ = (β-α)/60) value of the coordination polyhedron, <τ> = 0.337 for Zn1 and <τ> = 0.191 for Zn2 which can be compared with the ideal value for 1 for trigonal-bipyramidal and 0 for square-pyramidal (Addison *et al.*, 1984), the environment of the metal center can be determinate. Each zinc center has a square pyramidal geometry with appreciable distortions as shown by the Addison <τ> parameter. The basal plane for earch Zn^II^ center is respectively constructed by the coordination of two nitrogen atoms and one oxygen atom from the organic ligand molecule and one chloride atom acting as monodentate anion to zinc. The fifth coordination site is completed by a monodentate chloride anion.

### Experimental

In a 50 ml round bottom flask introduce isonicotinic hydrazide (1 g, 0.0073 mol) dissolved in methanol (10 ml). Benzoylpyridine (1.35 g, 0.0073 mol) in methanol (10 ml) and two drops of galcial acetic acid, were added. The mixture was refluxed for six hours to yield a quantitative precipitate. The white precipitate formed, was separated by filtration, washed with ether and dried under vacuum (yield: 75.2%); m.p.=206 °C. ^1^H NMR in C_2_D_6_SO, δ (p.p.m.): 7.44–7.87 (m, 13H, H_Ar_),, 8.82 (s, 1H, –NH). ^13^C NMR in C_2_D_6_SO, δ (p.p.m.): 121–149 (C_Ar_), 152 (C=N), 162 (C=O). IR (cm^-1^) 3342, 3279, 3161, 1726, 1698, 1515, 1398, 1359, 1169, 787. Anal. Calc. for C_18_H_14_N_4_O (%): C, 71.51; H, 4.67; N, 18.53. Found: C, 71.53; H, 4.68; N, 18.57. Into an ethanolic solution (10 ml) of zinc chloride (0.067 g, 0.005 mmol) was added an ethanolic solution (10 ml) of the ligand prepared above (0.15 g, 0.005 mmol). The resulting yellow mixture is heated at 60°C for two hours. The yellow precipitate was discarded and the resulting filtrate was then allowed to evaporate slowly in an open atmosphere. After two days, yellow crystals suitable for X-ray analysis were obtained. The crystals were separated and dried (yield: 73%); Anal. Calc. for C_18_H_14_ZnCl_2_N_4_O (%): C, 49.29; H, 3.22; N, 12.77. Found: C, 49.32; H, 3.20; N, 12.75. Selected IR data (cm^-1^, KBr pellet): 3445, 3053, 1650, 1511, 1352, 1141, 700.

### Refinement

Six low-resolution reflections affected by the beamstop were omitted from the refinement using the *OMIT* instruction in *SHELXL97* (Sheldrick, 2008). All H atoms were initially located in difference Fourier maps, but for the final refinements, all benzene-bound H atoms were positioned with idealized geometry and included in the calculations as riding on their parent atoms, with C—H = 0.97 Å and *U*_iso_(H) = 1.2*U*_eq_(C). The positions of the H atoms borne by nitrogen atoms were refined freely, giving restraints on N—H distances in the range 0.87 (2) Å with *U*_iso_(H) = 1.2*U*_eq_(N).

### Crystal data

**Table:**

[ZnCl_2_(C_18_H_14_N_4_O)]	*Z* = 4
*M**_r_* = 438.6	*F*(000) = 888
Triclinic, *P*1	*D*_x_ = 1.569 Mg m^−^^3^
Hall symbol: -P 1	Mo *K*α radiation, λ = 0.71070 Å
*a* = 11.517 (3) Å	Cell parameters from 5145 reflections
*b* = 13.248 (2) Å	θ = 0.4–24.7°
*c* = 13.459 (1) Å	µ = 1.63 mm^−^^1^
α = 91.130 (5)°	*T* = 293 K
β = 104.708 (4)°	Prism, yellow
γ = 109.798 (4)°	0.45 × 0.22 × 0.18 mm
*V* = 1856.2 (6) Å^3^	

### Data collection

**Table:**

Nonius KappaCCD diffractometer	6674 independent reflections
Radiation source: fine-focus sealed tube, Nonius KappaCCD	4122 reflections with *I* > 2σ(*I*)
Graphite monochromator	*R*_int_ = 0.040
Detector resolution: 9 pixels mm^-1^	θ_max_ = 25.3°, θ_min_ = 1.6°
phi and ω scans	*h* = −13→13
Absorption correction: multi-scan (*ABSCOR*; Rigaku, 2009)	*k* = −15→15
*T*_min_ = 0.379, *T*_max_ = 0.745	*l* = −16→16
15524 measured reflections	

### Refinement

**Table:**

Refinement on *F*^2^	Secondary atom site location: difference Fourier map
Least-squares matrix: full	Hydrogen site location: difference Fourier map
*R*[*F*^2^ > 2σ(*F*^2^)] = 0.042	H atoms treated by a mixture of independent and constrained refinement
*wR*(*F*^2^) = 0.102	*w* = 1/[σ^2^(*F*_o_^2^) + (0.0372*P*)^2^] where *P* = (*F*_o_^2^ + 2*F*_c_^2^)/3
*S* = 0.97	(Δ/σ)_max_ = 0.001
6668 reflections	Δρ_max_ = 0.32 e Å^−^^3^
476 parameters	Δρ_min_ = −0.42 e Å^−^^3^
1 restraint	Extinction correction: *SHELXL97* (Sheldrick, 2008), Fc^*^=kFc[1+0.001xFc^2^λ^3^/sin(2θ)]^-1/4^
Primary atom site location: structure-invariant direct methods	Extinction coefficient: 0.0013 (3)

### Special details

**Table:**

Geometry. All e.s.d.'s (except the e.s.d. in the dihedral angle between two l.s. planes) are estimated using the full covariance matrix. The cell e.s.d.'s are taken into account individually in the estimation of e.s.d.'s in distances, angles and torsion angles; correlations between e.s.d.'s in cell parameters are only used when they are defined by crystal symmetry. An approximate (isotropic) treatment of cell e.s.d.'s is used for estimating e.s.d.'s involving l.s. planes.
Refinement. Refinement of *F*^2^ against ALL reflections. The weighted *R*-factor *wR* and goodness of fit *S* are based on *F*^2^, conventional *R*-factors *R* are based on *F*, with *F* set to zero for negative *F*^2^. The threshold expression of *F*^2^ > σ(*F*^2^) is used only for calculating *R*-factors(gt) *etc*. and is not relevant to the choice of reflections for refinement. *R*-factors based on *F*^2^ are statistically about twice as large as those based on *F*, and *R*- factors based on ALL data will be even larger. Six low-resolution reflections were rejected due to beamstop shading using the *OMIT* instruction from *SHELX97*-*L*.

### Fractional atomic coordinates and isotropic or equivalent isotropic displacement parameters (Å^2^)

**Table:**

	*x*	*y*	*z*	*U*_iso_*/*U*_eq_	
Zn1	0.17339 (4)	0.03172 (4)	0.30977 (3)	0.04231 (15)	
Cl1	0.13436 (11)	−0.14621 (9)	0.29370 (9)	0.0661 (3)	
Cl2	0.00008 (9)	0.07571 (9)	0.31555 (8)	0.0552 (3)	
O1	0.1627 (3)	0.0699 (3)	0.15571 (19)	0.0685 (10)	
N1	0.3803 (3)	0.1689 (3)	0.2234 (2)	0.0429 (8)	
N2	0.3573 (3)	0.1346 (2)	0.3148 (2)	0.0404 (8)	
N3	0.2819 (3)	0.0655 (2)	0.4742 (2)	0.0393 (8)	
N4	0.2859 (3)	0.2290 (3)	−0.1453 (2)	0.0488 (9)	
H4N	0.288 (3)	0.247 (3)	−0.215 (3)	0.059*	
C1	0.2704 (4)	0.1297 (3)	0.1489 (3)	0.0416 (10)	
C2	0.4498 (3)	0.1642 (3)	0.4008 (3)	0.0369 (9)	
C3	0.4088 (3)	0.1201 (3)	0.4924 (2)	0.0356 (9)	
C4	0.4913 (4)	0.1357 (3)	0.5905 (3)	0.0479 (11)	
H4	0.5785	0.1750	0.6026	0.058*	
C5	0.4432 (4)	0.0924 (3)	0.6705 (3)	0.0540 (12)	
H5	0.4982	0.1001	0.7364	0.065*	
C6	0.3148 (4)	0.0384 (3)	0.6523 (3)	0.0548 (12)	
H6	0.2805	0.0101	0.7056	0.066*	
C7	0.2368 (4)	0.0265 (3)	0.5531 (3)	0.0482 (11)	
H7	0.1490	−0.0103	0.5406	0.058*	
C8	0.2787 (3)	0.1648 (3)	0.0446 (2)	0.0378 (9)	
C9	0.1685 (4)	0.1343 (3)	−0.0369 (3)	0.0510 (11)	
H9	0.0903	0.0911	−0.0278	0.061*	
C10	0.1756 (4)	0.1681 (3)	−0.1311 (3)	0.0529 (12)	
H10	0.1014	0.1479	−0.1861	0.064*	
C11	0.3953 (4)	0.2585 (3)	−0.0685 (3)	0.0514 (11)	
H11	0.4725	0.3001	−0.0805	0.062*	
C12	0.3932 (4)	0.2275 (3)	0.0277 (3)	0.0437 (10)	
H12	0.4688	0.2485	0.0814	0.052*	
C13	0.5813 (3)	0.2388 (3)	0.4099 (2)	0.0361 (9)	
C14	0.6004 (4)	0.3387 (3)	0.3735 (3)	0.0447 (10)	
H14	0.5298	0.3568	0.3408	0.054*	
C15	0.7222 (4)	0.4112 (4)	0.3850 (3)	0.0566 (12)	
H15	0.7344	0.4785	0.3614	0.068*	
C16	0.8262 (4)	0.3831 (5)	0.4321 (3)	0.0732 (16)	
H16	0.9090	0.4312	0.4392	0.088*	
C17	0.8084 (4)	0.2860 (5)	0.4682 (4)	0.0765 (16)	
H17	0.8793	0.2681	0.5003	0.092*	
C18	0.6868 (4)	0.2134 (4)	0.4579 (3)	0.0539 (11)	
H18	0.6759	0.1471	0.4834	0.065*	
Zn2	0.31778 (4)	0.47927 (4)	0.17144 (3)	0.04310 (16)	
Cl3	0.50465 (9)	0.47545 (9)	0.15904 (7)	0.0536 (3)	
Cl4	0.32363 (11)	0.64860 (9)	0.19446 (9)	0.0688 (4)	
O2	0.3419 (2)	0.4448 (2)	0.33683 (18)	0.0530 (8)	
N5	0.1412 (3)	0.3230 (3)	0.2757 (2)	0.0439 (8)	
H5N	0.085 (3)	0.265 (2)	0.283 (3)	0.053*	
N6	0.1446 (3)	0.3573 (2)	0.1811 (2)	0.0414 (8)	
N7	0.1966 (3)	0.4295 (3)	0.0172 (2)	0.0460 (9)	
N8	0.2668 (3)	0.2860 (3)	0.6574 (2)	0.0478 (9)	
C19	0.2514 (3)	0.3738 (3)	0.3533 (3)	0.0406 (10)	
C20	0.0454 (3)	0.3233 (3)	0.1017 (3)	0.0375 (9)	
C21	0.0725 (3)	0.3694 (3)	0.0067 (2)	0.0365 (9)	
C22	−0.0200 (4)	0.3560 (3)	−0.0847 (3)	0.0468 (11)	
H22	−0.1059	0.3176	−0.0899	0.056*	
C23	0.0162 (4)	0.4007 (4)	−0.1695 (3)	0.0605 (13)	
H23	−0.0451	0.3914	−0.2325	0.073*	
C24	0.1421 (4)	0.4581 (4)	−0.1598 (3)	0.0611 (13)	
H24	0.1685	0.4873	−0.2161	0.073*	
C25	0.2298 (4)	0.4721 (4)	−0.0645 (3)	0.0581 (12)	
H25	0.3156	0.5130	−0.0571	0.070*	
C26	0.2530 (3)	0.3413 (3)	0.4580 (2)	0.0360 (9)	
C27	0.3693 (3)	0.3604 (3)	0.5289 (3)	0.0395 (9)	
H27	0.4454	0.3924	0.5111	0.047*	
C28	0.3718 (4)	0.3316 (3)	0.6264 (3)	0.0451 (10)	
H28	0.4513	0.3446	0.6736	0.054*	
C29	0.1532 (4)	0.2681 (3)	0.5884 (3)	0.0477 (11)	
H29	0.0786	0.2362	0.6085	0.057*	
C30	0.1422 (3)	0.2949 (3)	0.4894 (3)	0.0433 (10)	
H30	0.0617	0.2822	0.4438	0.052*	
C31	−0.0845 (3)	0.2501 (3)	0.1009 (3)	0.0396 (9)	
C32	−0.1420 (4)	0.2719 (3)	0.1729 (3)	0.0484 (11)	
H32	−0.0964	0.3307	0.2233	0.058*	
C33	−0.2659 (4)	0.2085 (4)	0.1718 (4)	0.0658 (13)	
H33	−0.3040	0.2247	0.2202	0.079*	
C34	−0.3308 (4)	0.1214 (4)	0.0979 (4)	0.0699 (14)	
H34	−0.4140	0.0778	0.0962	0.084*	
C35	−0.2754 (4)	0.0976 (4)	0.0266 (4)	0.0708 (14)	
H35	−0.3213	0.0381	−0.0231	0.085*	
C36	−0.1524 (4)	0.1606 (3)	0.0274 (3)	0.0572 (12)	
H36	−0.1150	0.1434	−0.0210	0.069*	

### Atomic displacement parameters (Å^2^)

**Table:**

	*U*^11^	*U*^22^	*U*^33^	*U*^12^	*U*^13^	*U*^23^
Zn1	0.0348 (3)	0.0483 (3)	0.0352 (2)	0.0049 (2)	0.00822 (19)	0.0085 (2)
Cl1	0.0643 (7)	0.0521 (7)	0.0679 (7)	0.0167 (6)	0.0003 (6)	0.0003 (6)
Cl2	0.0417 (6)	0.0625 (7)	0.0589 (6)	0.0144 (5)	0.0150 (5)	0.0116 (5)
O1	0.0372 (16)	0.108 (3)	0.0367 (15)	−0.0019 (17)	0.0071 (12)	0.0231 (16)
N1	0.0378 (18)	0.055 (2)	0.0263 (15)	0.0062 (16)	0.0066 (13)	0.0109 (14)
N2	0.0388 (18)	0.0483 (19)	0.0290 (15)	0.0085 (15)	0.0098 (14)	0.0108 (14)
N3	0.0416 (19)	0.0428 (19)	0.0333 (16)	0.0112 (15)	0.0146 (14)	0.0098 (14)
N4	0.055 (2)	0.055 (2)	0.0299 (17)	0.0147 (19)	0.0083 (16)	0.0119 (16)
C1	0.043 (2)	0.048 (2)	0.0304 (19)	0.012 (2)	0.0078 (18)	0.0131 (17)
C2	0.039 (2)	0.036 (2)	0.0320 (19)	0.0091 (18)	0.0088 (17)	0.0089 (16)
C3	0.035 (2)	0.036 (2)	0.0319 (19)	0.0088 (18)	0.0090 (16)	0.0079 (16)
C4	0.041 (2)	0.054 (3)	0.036 (2)	0.005 (2)	0.0056 (18)	0.0066 (19)
C5	0.058 (3)	0.067 (3)	0.032 (2)	0.018 (2)	0.0091 (19)	0.017 (2)
C6	0.064 (3)	0.065 (3)	0.036 (2)	0.020 (2)	0.019 (2)	0.014 (2)
C7	0.045 (2)	0.052 (3)	0.043 (2)	0.008 (2)	0.0153 (19)	0.016 (2)
C8	0.037 (2)	0.041 (2)	0.0318 (19)	0.0103 (18)	0.0076 (16)	0.0081 (17)
C9	0.041 (2)	0.064 (3)	0.037 (2)	0.008 (2)	0.0048 (18)	0.015 (2)
C10	0.052 (3)	0.063 (3)	0.033 (2)	0.015 (2)	0.0000 (19)	0.010 (2)
C11	0.048 (2)	0.057 (3)	0.040 (2)	0.005 (2)	0.0127 (19)	0.013 (2)
C12	0.042 (2)	0.053 (3)	0.0266 (18)	0.010 (2)	0.0039 (16)	0.0089 (18)
C13	0.032 (2)	0.044 (2)	0.0294 (18)	0.0130 (18)	0.0059 (16)	0.0037 (17)
C14	0.041 (2)	0.052 (3)	0.040 (2)	0.014 (2)	0.0113 (18)	0.0108 (19)
C15	0.053 (3)	0.054 (3)	0.054 (2)	0.003 (2)	0.021 (2)	0.007 (2)
C16	0.039 (3)	0.091 (4)	0.061 (3)	−0.011 (3)	0.011 (2)	0.009 (3)
C17	0.031 (2)	0.127 (5)	0.071 (3)	0.027 (3)	0.012 (2)	0.028 (3)
C18	0.051 (3)	0.070 (3)	0.052 (2)	0.030 (2)	0.019 (2)	0.027 (2)
Zn2	0.0313 (2)	0.0511 (3)	0.0382 (3)	0.0049 (2)	0.00794 (19)	0.0067 (2)
Cl3	0.0450 (6)	0.0684 (7)	0.0499 (6)	0.0215 (5)	0.0158 (5)	0.0086 (5)
Cl4	0.0511 (7)	0.0624 (7)	0.0814 (8)	0.0248 (6)	−0.0065 (6)	−0.0050 (6)
O2	0.0337 (15)	0.0702 (19)	0.0363 (14)	−0.0019 (14)	0.0052 (12)	0.0095 (13)
N5	0.0336 (18)	0.056 (2)	0.0309 (16)	0.0033 (16)	0.0063 (14)	0.0119 (16)
N6	0.0359 (17)	0.053 (2)	0.0280 (15)	0.0086 (16)	0.0051 (13)	0.0090 (14)
N7	0.0383 (18)	0.053 (2)	0.0368 (17)	0.0035 (16)	0.0111 (14)	0.0088 (15)
N8	0.049 (2)	0.054 (2)	0.0324 (16)	0.0127 (17)	0.0038 (16)	0.0106 (15)
C19	0.031 (2)	0.056 (3)	0.0318 (19)	0.013 (2)	0.0073 (16)	0.0049 (18)
C20	0.031 (2)	0.043 (2)	0.0341 (19)	0.0083 (18)	0.0078 (16)	0.0036 (17)
C21	0.037 (2)	0.037 (2)	0.0344 (19)	0.0115 (18)	0.0089 (17)	0.0053 (17)
C22	0.041 (2)	0.057 (3)	0.036 (2)	0.014 (2)	0.0030 (18)	0.0120 (19)
C23	0.064 (3)	0.070 (3)	0.038 (2)	0.020 (3)	0.003 (2)	0.010 (2)
C24	0.077 (3)	0.073 (3)	0.034 (2)	0.024 (3)	0.021 (2)	0.022 (2)
C25	0.052 (3)	0.072 (3)	0.045 (2)	0.012 (2)	0.020 (2)	0.013 (2)
C26	0.034 (2)	0.042 (2)	0.0296 (18)	0.0112 (17)	0.0073 (16)	0.0049 (16)
C27	0.031 (2)	0.048 (2)	0.0334 (19)	0.0101 (18)	0.0045 (16)	0.0069 (17)
C28	0.041 (2)	0.054 (3)	0.031 (2)	0.015 (2)	−0.0027 (18)	−0.0009 (18)
C29	0.044 (2)	0.061 (3)	0.035 (2)	0.012 (2)	0.0147 (18)	0.0110 (19)
C30	0.030 (2)	0.057 (3)	0.036 (2)	0.0110 (19)	0.0046 (17)	0.0082 (19)
C31	0.030 (2)	0.045 (2)	0.037 (2)	0.0095 (18)	0.0037 (17)	0.0095 (18)
C32	0.042 (2)	0.055 (3)	0.042 (2)	0.012 (2)	0.0095 (19)	0.0108 (19)
C33	0.042 (3)	0.088 (4)	0.069 (3)	0.019 (3)	0.022 (2)	0.017 (3)
C34	0.035 (2)	0.066 (3)	0.094 (4)	0.002 (2)	0.014 (3)	0.014 (3)
C35	0.048 (3)	0.052 (3)	0.095 (4)	0.005 (2)	0.007 (3)	−0.006 (3)
C36	0.041 (2)	0.050 (3)	0.068 (3)	0.010 (2)	0.004 (2)	−0.009 (2)

### Geometric parameters (Å, º)

**Table:**

Zn1—N2	2.078 (3)	Zn2—N7	2.131 (3)
Zn1—O1	2.127 (3)	Zn2—N6	2.136 (3)
Zn1—N3	2.200 (3)	Zn2—Cl3	2.2176 (13)
Zn1—Cl1	2.2400 (13)	Zn2—Cl4	2.2343 (13)
Zn1—Cl2	2.2793 (13)	Zn2—O2	2.248 (2)
O1—C1	1.252 (4)	O2—C19	1.217 (4)
N1—C1	1.330 (4)	N5—N6	1.367 (4)
N1—N2	1.377 (4)	N5—C19	1.369 (4)
N2—C2	1.304 (4)	N5—H5N	0.849 (18)
N3—C7	1.336 (4)	N6—C20	1.289 (4)
N3—C3	1.348 (4)	N7—C25	1.329 (5)
N4—C10	1.318 (5)	N7—C21	1.348 (4)
N4—C11	1.341 (5)	N8—C28	1.332 (5)
N4—H4N	0.98 (4)	N8—C29	1.340 (5)
C1—C8	1.503 (5)	C19—C26	1.479 (5)
C2—C13	1.474 (5)	C20—C31	1.479 (5)
C2—C3	1.490 (5)	C20—C21	1.487 (5)
C3—C4	1.381 (4)	C21—C22	1.370 (5)
C4—C5	1.382 (5)	C22—C23	1.389 (5)
C4—H4	0.9300	C22—H22	0.9300
C5—C6	1.360 (5)	C23—C24	1.360 (6)
C5—H5	0.9300	C23—H23	0.9300
C6—C7	1.379 (5)	C24—C25	1.381 (5)
C6—H6	0.9300	C24—H24	0.9300
C7—H7	0.9300	C25—H25	0.9300
C8—C12	1.378 (5)	C26—C27	1.372 (5)
C8—C9	1.380 (5)	C26—C30	1.392 (5)
C9—C10	1.366 (5)	C27—C28	1.370 (5)
C9—H9	0.9300	C27—H27	0.9300
C10—H10	0.9300	C28—H28	0.9300
C11—C12	1.370 (5)	C29—C30	1.372 (5)
C11—H11	0.9300	C29—H29	0.9300
C12—H12	0.9300	C30—H30	0.9300
C13—C18	1.377 (5)	C31—C32	1.379 (5)
C13—C14	1.387 (5)	C31—C36	1.389 (5)
C14—C15	1.373 (5)	C32—C33	1.385 (5)
C14—H14	0.9300	C32—H32	0.9300
C15—C16	1.379 (7)	C33—C34	1.369 (6)
C15—H15	0.9300	C33—H33	0.9300
C16—C17	1.352 (7)	C34—C35	1.364 (6)
C16—H16	0.9300	C34—H34	0.9300
C17—C18	1.374 (6)	C35—C36	1.377 (6)
C17—H17	0.9300	C35—H35	0.9300
C18—H18	0.9300	C36—H36	0.9300
			
N2—Zn1—O1	74.86 (10)	N7—Zn2—N6	73.37 (11)
N2—Zn1—N3	74.29 (11)	N7—Zn2—Cl3	103.43 (10)
O1—Zn1—N3	148.50 (11)	N6—Zn2—Cl3	133.30 (10)
N2—Zn1—Cl1	117.50 (10)	N7—Zn2—Cl4	100.89 (10)
O1—Zn1—Cl1	102.71 (10)	N6—Zn2—Cl4	114.89 (10)
N3—Zn1—Cl1	97.02 (9)	Cl3—Zn2—Cl4	111.46 (5)
N2—Zn1—Cl2	128.30 (10)	N7—Zn2—O2	144.79 (11)
O1—Zn1—Cl2	93.30 (9)	N6—Zn2—O2	71.72 (9)
N3—Zn1—Cl2	100.75 (9)	Cl3—Zn2—O2	97.10 (8)
Cl1—Zn1—Cl2	114.20 (5)	Cl4—Zn2—O2	97.70 (8)
C1—O1—Zn1	110.7 (2)	C19—O2—Zn2	115.5 (2)
C1—N1—N2	108.2 (3)	N6—N5—C19	114.4 (3)
C2—N2—N1	120.4 (3)	N6—N5—H5N	122 (3)
C2—N2—Zn1	121.7 (2)	C19—N5—H5N	122 (3)
N1—N2—Zn1	117.9 (2)	C20—N6—N5	121.9 (3)
C7—N3—C3	118.7 (3)	C20—N6—Zn2	120.7 (2)
C7—N3—Zn1	126.2 (2)	N5—N6—Zn2	117.3 (2)
C3—N3—Zn1	114.6 (2)	C25—N7—C21	118.9 (3)
C10—N4—C11	121.3 (3)	C25—N7—Zn2	123.1 (3)
C10—N4—H4N	118 (2)	C21—N7—Zn2	116.5 (2)
C11—N4—H4N	120 (2)	C28—N8—C29	117.3 (3)
O1—C1—N1	128.2 (3)	O2—C19—N5	121.1 (3)
O1—C1—C8	117.2 (3)	O2—C19—C26	122.0 (3)
N1—C1—C8	114.5 (3)	N5—C19—C26	116.9 (3)
N2—C2—C13	124.3 (3)	N6—C20—C31	126.2 (3)
N2—C2—C3	113.6 (3)	N6—C20—C21	112.5 (3)
C13—C2—C3	122.0 (3)	C31—C20—C21	121.3 (3)
N3—C3—C4	121.0 (3)	N7—C21—C22	121.3 (3)
N3—C3—C2	115.0 (3)	N7—C21—C20	114.8 (3)
C4—C3—C2	124.0 (3)	C22—C21—C20	123.8 (3)
C3—C4—C5	119.4 (4)	C21—C22—C23	119.1 (4)
C3—C4—H4	120.3	C21—C22—H22	120.4
C5—C4—H4	120.3	C23—C22—H22	120.4
C6—C5—C4	119.6 (3)	C24—C23—C22	119.5 (4)
C6—C5—H5	120.2	C24—C23—H23	120.3
C4—C5—H5	120.2	C22—C23—H23	120.3
C5—C6—C7	118.5 (4)	C23—C24—C25	118.5 (4)
C5—C6—H6	120.8	C23—C24—H24	120.7
C7—C6—H6	120.8	C25—C24—H24	120.7
N3—C7—C6	122.9 (4)	N7—C25—C24	122.6 (4)
N3—C7—H7	118.6	N7—C25—H25	118.7
C6—C7—H7	118.6	C24—C25—H25	118.7
C12—C8—C9	118.6 (3)	C27—C26—C30	118.1 (3)
C12—C8—C1	122.0 (3)	C27—C26—C19	118.6 (3)
C9—C8—C1	119.3 (3)	C30—C26—C19	123.3 (3)
C10—C9—C8	119.4 (4)	C28—C27—C26	119.0 (4)
C10—C9—H9	120.3	C28—C27—H27	120.5
C8—C9—H9	120.3	C26—C27—H27	120.5
N4—C10—C9	121.0 (4)	N8—C28—C27	123.7 (3)
N4—C10—H10	119.5	N8—C28—H28	118.2
C9—C10—H10	119.5	C27—C28—H28	118.2
N4—C11—C12	120.0 (4)	N8—C29—C30	122.7 (4)
N4—C11—H11	120.0	N8—C29—H29	118.6
C12—C11—H11	120.0	C30—C29—H29	118.6
C11—C12—C8	119.7 (3)	C29—C30—C26	119.1 (3)
C11—C12—H12	120.2	C29—C30—H30	120.4
C8—C12—H12	120.2	C26—C30—H30	120.4
C18—C13—C14	118.8 (4)	C32—C31—C36	118.7 (4)
C18—C13—C2	121.3 (4)	C32—C31—C20	119.8 (3)
C14—C13—C2	119.8 (3)	C36—C31—C20	121.5 (4)
C15—C14—C13	120.8 (4)	C31—C32—C33	121.7 (4)
C15—C14—H14	119.6	C31—C32—H32	119.1
C13—C14—H14	119.6	C33—C32—H32	119.1
C14—C15—C16	119.2 (4)	C34—C33—C32	118.3 (4)
C14—C15—H15	120.4	C34—C33—H33	120.8
C16—C15—H15	120.4	C32—C33—H33	120.8
C17—C16—C15	120.4 (4)	C35—C34—C33	121.0 (4)
C17—C16—H16	119.8	C35—C34—H34	119.5
C15—C16—H16	119.8	C33—C34—H34	119.5
C16—C17—C18	120.7 (4)	C34—C35—C36	120.8 (4)
C16—C17—H17	119.6	C34—C35—H35	119.6
C18—C17—H17	119.6	C36—C35—H35	119.6
C17—C18—C13	120.1 (4)	C35—C36—C31	119.5 (4)
C17—C18—H18	120.0	C35—C36—H36	120.2
C13—C18—H18	120.0	C31—C36—H36	120.2
			
N2—Zn1—O1—C1	2.0 (3)	N7—Zn2—O2—C19	−5.4 (4)
N3—Zn1—O1—C1	14.0 (5)	N6—Zn2—O2—C19	2.3 (3)
Cl1—Zn1—O1—C1	−113.5 (3)	Cl3—Zn2—O2—C19	−131.1 (3)
Cl2—Zn1—O1—C1	130.8 (3)	Cl4—Zn2—O2—C19	116.0 (3)
C1—N1—N2—C2	−178.5 (3)	C19—N5—N6—C20	−174.8 (4)
C1—N1—N2—Zn1	3.3 (4)	C19—N5—N6—Zn2	0.8 (4)
O1—Zn1—N2—C2	178.9 (3)	N7—Zn2—N6—C20	−10.5 (3)
N3—Zn1—N2—C2	5.3 (3)	Cl3—Zn2—N6—C20	−103.5 (3)
Cl1—Zn1—N2—C2	−84.5 (3)	Cl4—Zn2—N6—C20	84.0 (3)
Cl2—Zn1—N2—C2	96.8 (3)	O2—Zn2—N6—C20	174.1 (3)
O1—Zn1—N2—N1	−2.9 (3)	N7—Zn2—N6—N5	173.8 (3)
N3—Zn1—N2—N1	−176.5 (3)	Cl3—Zn2—N6—N5	80.9 (3)
Cl1—Zn1—N2—N1	93.7 (3)	Cl4—Zn2—N6—N5	−91.7 (3)
Cl2—Zn1—N2—N1	−85.0 (3)	O2—Zn2—N6—N5	−1.5 (3)
N2—Zn1—N3—C7	−179.7 (4)	N6—Zn2—N7—C25	179.1 (4)
O1—Zn1—N3—C7	168.2 (3)	Cl3—Zn2—N7—C25	−49.3 (4)
Cl1—Zn1—N3—C7	−63.1 (3)	Cl4—Zn2—N7—C25	66.1 (4)
Cl2—Zn1—N3—C7	53.3 (3)	O2—Zn2—N7—C25	−173.3 (3)
N2—Zn1—N3—C3	−8.2 (3)	N6—Zn2—N7—C21	13.3 (3)
O1—Zn1—N3—C3	−20.2 (4)	Cl3—Zn2—N7—C21	145.0 (3)
Cl1—Zn1—N3—C3	108.5 (3)	Cl4—Zn2—N7—C21	−99.6 (3)
Cl2—Zn1—N3—C3	−135.2 (3)	O2—Zn2—N7—C21	21.0 (4)
Zn1—O1—C1—N1	−0.9 (6)	Zn2—O2—C19—N5	−2.8 (5)
Zn1—O1—C1—C8	−179.5 (3)	Zn2—O2—C19—C26	179.6 (3)
N2—N1—C1—O1	−1.5 (6)	N6—N5—C19—O2	1.4 (6)
N2—N1—C1—C8	177.2 (3)	N6—N5—C19—C26	179.1 (3)
N1—N2—C2—C13	3.3 (6)	N5—N6—C20—C31	3.7 (6)
Zn1—N2—C2—C13	−178.5 (3)	Zn2—N6—C20—C31	−171.7 (3)
N1—N2—C2—C3	−180.0 (3)	N5—N6—C20—C21	−178.5 (3)
Zn1—N2—C2—C3	−1.8 (4)	Zn2—N6—C20—C21	6.0 (4)
C7—N3—C3—C4	0.1 (6)	C25—N7—C21—C22	−3.2 (6)
Zn1—N3—C3—C4	−172.2 (3)	Zn2—N7—C21—C22	163.2 (3)
C7—N3—C3—C2	−177.8 (3)	C25—N7—C21—C20	178.7 (4)
Zn1—N3—C3—C2	10.0 (4)	Zn2—N7—C21—C20	−14.9 (4)
N2—C2—C3—N3	−5.8 (5)	N6—C20—C21—N7	5.9 (5)
C13—C2—C3—N3	171.1 (3)	C31—C20—C21—N7	−176.2 (3)
N2—C2—C3—C4	176.5 (4)	N6—C20—C21—C22	−172.1 (4)
C13—C2—C3—C4	−6.7 (6)	C31—C20—C21—C22	5.7 (6)
N3—C3—C4—C5	1.5 (6)	N7—C21—C22—C23	3.4 (6)
C2—C3—C4—C5	179.2 (4)	C20—C21—C22—C23	−178.6 (4)
C3—C4—C5—C6	−2.3 (7)	C21—C22—C23—C24	−1.1 (7)
C4—C5—C6—C7	1.5 (7)	C22—C23—C24—C25	−1.4 (7)
C3—N3—C7—C6	−0.9 (6)	C21—N7—C25—C24	0.6 (7)
Zn1—N3—C7—C6	170.3 (3)	Zn2—N7—C25—C24	−164.8 (3)
C5—C6—C7—N3	0.1 (7)	C23—C24—C25—N7	1.7 (7)
O1—C1—C8—C12	−177.7 (4)	O2—C19—C26—C27	−24.1 (6)
N1—C1—C8—C12	3.5 (6)	N5—C19—C26—C27	158.2 (4)
O1—C1—C8—C9	2.7 (6)	O2—C19—C26—C30	154.0 (4)
N1—C1—C8—C9	−176.1 (4)	N5—C19—C26—C30	−23.6 (6)
C12—C8—C9—C10	−1.1 (6)	C30—C26—C27—C28	1.1 (6)
C1—C8—C9—C10	178.6 (4)	C19—C26—C27—C28	179.3 (3)
C11—N4—C10—C9	1.0 (6)	C29—N8—C28—C27	−0.1 (6)
C8—C9—C10—N4	0.3 (7)	C26—C27—C28—N8	−0.4 (6)
C10—N4—C11—C12	−1.5 (6)	C28—N8—C29—C30	−0.1 (6)
N4—C11—C12—C8	0.7 (6)	N8—C29—C30—C26	0.9 (6)
C9—C8—C12—C11	0.5 (6)	C27—C26—C30—C29	−1.3 (6)
C1—C8—C12—C11	−179.1 (4)	C19—C26—C30—C29	−179.5 (4)
N2—C2—C13—C18	−130.1 (4)	N6—C20—C31—C32	50.0 (6)
C3—C2—C13—C18	53.5 (5)	C21—C20—C31—C32	−127.6 (4)
N2—C2—C13—C14	52.6 (5)	N6—C20—C31—C36	−131.8 (4)
C3—C2—C13—C14	−123.9 (4)	C21—C20—C31—C36	50.6 (5)
C18—C13—C14—C15	−0.1 (6)	C36—C31—C32—C33	−1.5 (6)
C2—C13—C14—C15	177.3 (3)	C20—C31—C32—C33	176.8 (4)
C13—C14—C15—C16	1.0 (6)	C31—C32—C33—C34	0.9 (7)
C14—C15—C16—C17	−1.2 (7)	C32—C33—C34—C35	−0.2 (7)
C15—C16—C17—C18	0.5 (8)	C33—C34—C35—C36	0.1 (8)
C16—C17—C18—C13	0.4 (7)	C34—C35—C36—C31	−0.7 (7)
C14—C13—C18—C17	−0.6 (6)	C32—C31—C36—C35	1.3 (6)
C2—C13—C18—C17	−177.9 (4)	C20—C31—C36—C35	−177.0 (4)

### Hydrogen-bond geometry (Å, º)

**Table:**

*D*—H···*A*	*D*—H	H···*A*	*D*···*A*	*D*—H···*A*
N5—H5*N*···Cl2	0.85 (2)	2.46 (2)	3.250 (3)	156 (4)
N4—H4*N*···N8^i^	0.98 (4)	1.78 (4)	2.749 (4)	170 (4)

Symmetry code: (i) *x*, *y*, *z*−1.
